# Up-regulation of P21-activated kinase 1 in osteoarthritis chondrocytes is responsible for osteoarthritic cartilage destruction

**DOI:** 10.1042/BSR20191017

**Published:** 2020-01-10

**Authors:** Wanli Ma, Xiaohe Wang, Chunhui Wang, Mingzhi Gong, Peng Ren

**Affiliations:** 1 Department of Orthopaedics, The Second Hospital of Shandong University, Jinan, P. R. China; 2 Department of Orthopaedics, Zhangqiu City Hospital of Traditional Chinese Medicine, Jinan, P. R. China; 3 Department of Orthopaedics, Licheng City Hospital of Traditional Chinese Medicine, Jinan, P. R. China

**Keywords:** Chondrocytes, Osteoarthritis, P21-activated Kinase-1, Proliferation

## Abstract

Osteoarthritis is mainly caused by a degenerative joint disorder, which is characterized by the gradual degradation of articular cartilage and synovial inflammation. The chondrocyte, the unique resident cell type of articular cartilage, is crucial for the development of osteoarthritis. Previous studies revealed that P21-activated kinase-1 (PAK1) was responsible for the initiation of inflammation. The purpose of the present study was to determine the potential role of PAK1 in osteoarthritis. The level of PAK1 expression was measured by Western blot and quantitative real-time PCR in articular cartilage from osteoarthritis model rats and patients with osteoarthritis. In addition, the functional role of aberrant PAK1 expression was detected in the chondrocytes. We found that the expression of PAK1 was significantly increased in chondrocytes treated with osteoarthritis-related factors. Increased expression of PAK1 was also observed in knee articular cartilage samples from patients with osteoarthritis and osteoarthritis model rats. PAK1 was found to inhibit chondrocytes proliferation and to promote the production of inflammatory cytokines in cartilages chondrocytes. Furthermore, we found that PAK1 modulated the production of extracellular matrix and cartilage degrading enzymes in chondrocytes. Results of the present studies demonstrated that PAK1 might play an important role in the pathogenesis of osteoarthritis.

## Introduction

Osteoarthritis (OA) is one of the most prevalent age-related degenerative joint disorders, which cause pain and joint dysfunctions in the affected patients [1,2]. The gradual degradation of articular cartilage and synovial inflammation are thought to be the main pathological event responsible for the joint destruction and during development of OA [3]. Growing evidences suggested that mechanical, genetic factors and inflammatory processes within joint tissues were confirmed to associate with the onset and progression of OA [4]. Although many risk factors are contributed to the pathogenesis of OA, the exact mechanisms of OA need to be further explored.

Articular cartilage covers the surface of a diarthrodial joint, which is composed of only one cell type, chondrocytes, enclosed in a self-synthetized extracellular matrix (ECM). Cartilage ECM is comprised mainly of collagen type II fibrils, and proteoglycans, which confer to the cartilage structural rigidity and protective resiliency [5]. The chondrocyte plays a central role in the degradation of ECM, which maintains the balance between synthesis and degradation of the cartilage ECM [6,7]. Chondrocytes as quiescent cells cannot divide under physiological conditions [8]. In addition, the ECM is neither innerved nor vascularized. Cells cannot be supplied to compensate for potential cellular loss. Therefore, phenotypic stability, anabolic/catabolic homeostasis, and survival of chondrocytes are crucial for the maintenance of proper articular cartilage [9].

P-21 activated kinases (PAKs) are serine-threonine kinases, which can be activated by the small guanosine triohosphte (GTP) binding proteins cell division cycle 42 and Rac1. PAKs are critical for regulating intracellular signaling and cellular functions [10,11]. The PAK isoforms identified in mammalian cells are characterized into group I (PAK1-3) and group II (PAK4-6). The first group of PAKs shares a high sequence homology and is highly evolutionarily conserved [12], in which PAK1 is the most abundantly expressed PAK isoforms in mammalian cells. Previous studies showed that PAK1 was recognized as an important regulator of the inflammatory response [10]. Furthermore, PAK1 could regulate cells physiology, including cells proliferation, apoptosis, and differentiation [13,14]. However, it was unclear whether PAK1 was involved in the pathogenesis of OA.

In the present study, we found that the expression of PAK1 was significantly increased in chondrocytes treated with OA-related factors. Increased expression of PAK1 was also observed in knee articular cartilage samples from patients with OA and OA model rats. PAK1 was found to inhibit chondrocytes proliferation and to promote the production of inflammatory cytokines in cartilages chondrocytes. Furthermore, we found that PAK1 modulated the production of ECM and cartilage degrading enzymes in chondrocytes. Results of the present studies demonstrated that PAK1 might play an important role in the pathogenesis of OA.

## Materials and methods

### Cartilage samples from OA models rats and patients with OA

The knee joint OA model was made by destabilization of the medial meniscus (DMM) surgery in adult male Sprague-Dawley (SD) rats (two groups and each one *n* = 8; 8-week old; mean body weight = 230 g). All animal experiments were approved by the Institutional Animal Care and Use Committee and the Animal Experimental Ethics Committee of the Shandong University. All procedures involving animal experiments were completed in laboratory animal center of Shandong University. Rats were housed in groups of four per cage under standard conditions with a 12-h light/dark cycle, with unlimited access to food and water. Under isoflurane anesthesia (3–5% sleep box induction maintained at 2% via mask inhalation), fur was removed and skin was prepared using povidone-iodine and alcohol in triplicate. Joint capsule was incised and the medial meniscotibial ligament was sectioned, as described previously [15]. In the sham operation group, the joint cavities were opened without any treatment. Perioperative buprenorphine (0.03 mg/kg) was administered twice per day out to 48 h post-operation. All rats were allowed to move freely in the cages after DMM surgery, which were killed by an overdose of carbon dioxide at 12 weeks after DMM surgery.

Knee cartilage samples were obtained from trauma donor who needed lower limb amputation (ages 60–76 years, *n* = 4) and patients with OA who underwent total knee arthroplasty (ages 62–87 years, *n* = 4). Ethical approval was obtained from The Second Hospital of Shandong University review board for human knee cartilage samples. Informed consent was obtained from all patients. Cartilage specimens were washed with sterile phosphate-buffered saline (PBS) and stained with India ink. Areas of cartilage with no staining were considered as unaffected cartilage samples. Areas of cartilage with intense staining were considered as damaged cartilage samples [16].

### Histological analysis

Cartilage specimens were processed, as described previously [17]. Samples were fixed in 4% paraformaldehyde, followed by decalcification in Ethylene Diamine Tetraacetic Acid (EDTA)-buffered saline solution (pH 7.4, 0.25 M). Tissue sections embedded in paraffin were then cut longitudinally to obtain 5 μm sections, which were then deparaffinized in toluene, and dehydrated in a graded series of ethanol. Immunohistochemistry was accomplished to test the expression and distribution of PAK1 in tissue sections embedded in paraffin following instructions of Histostain-Plus Kit (Invitrogen, Carlsbad, CA, U.S.A.). The evaluation of positive-staining chondrocytes was performed according to previous studies [18]. Histological changes and proteoglycans/collagen content were observed by hematoxylin–eosin (HE) staining and Safranin-O/Fast Green staining.

### Chondrocytes culture

Rat articular chondrocytes and human articular chondrocytes were prepared by enzymatic digestion of the cartilage, as described previously [19,20]. Briefly, cartilage tissues were finely cut into small pieces (∼1 mm^3^), and then digested with 0.2% type II collagenase (Sigma Chemical Co., St. Louis, MO, U.S.A.) at 37°C for 6 h. The primary cells were cultured in 25 ml cell culture plates (Corning Inc., Corning, NY, U.S.A.) filled with Dulbecco’s Modified Eagle’s Medium (DMEM, Gibco, Grand Island, NY, U.S.A.) supplemented with 10% fetal bovine serum (FBS, Gibco) and antibiotics (Invitrogen Corp., Carlsbad, CA, U.S.A.). Then, the cells were cultured under sterile conditions at 37°C in 5% CO_2_ incubator and used within the first three passages.

For mechanical stress stimulation, cells were subjected to mechanical stress with a 0.5 Hz sinusoidal curve at 10% elongation for 24 h using an Flexcell1 FX-5000™ Tension System, as described in the manufacturer’s manual (Flexcell International Corporation, Burlington, NC).

### Transfection of chondrocytes

PAK1 overexpression plasmid was made by Genechem (Shanghai, China). The siRNA duplexes against rat PAK1 were synthesized by GenePharma (Shanghai, China). The chondrocytes were seeded at a density of 1 × 10^6^ into a six-well tissue culture plate. When chondrocytes were grown to 80% confluence, cells were transfected with 100 nM siRNA PAK1 or 2 μg plasmid DNA for 24 h using Lipofectamine 3000 reagent (Invitrogen, Paisley, U.K.), according to the manufacturer’s protocols. Negative control (NC) cells were transduced with negative control-siRNA or mock plasmid.

### Immunofluorescence staining

Chondrocytes were fixed with 4% paraformaldehyde for 15 min, permeabilized with 0.5% Triton X-100 for 15 min, blocked with 10% goat serum in PBS for 30 min, and reacted with 1:200-diluted anti-PAK1 antibodies (Abcam, Cambridge, U.K.) in PBS for 2 h at room temperature. The cells were then washed with PBS and incubated with 1:1000-diluted secondary antibody (Abcam, Cambridge, U.K.) for 1 h at room temperature, and washed three times with PBS.

### Western blot analysis

The plasma protein of chondrocytes and articular cartilage were extracted in ice-cold lysis buffer. The plasma proteins concentration was determined using the Bicinchoninic acid (BCA) Protein Assay Kit (Pierce, Rockford, IL, U.S.A.). Twenty micrograms of total protein was subjected to sodium salt-polyacrylamide gel electrophoresis (SDS-PAGE) and electrotransferred onto a polyvinylidene difluoride membrane (0.45 mm; Millipore, Bedford, MA, U.S.A.). After blocking with 5% nonfat milk for 1 h, membranes were incubated overnight at 4°C with anti-pT423-PAK1 antibodies (diluted 1:1000, Cell Signaling Technology, catalog number:2601T), anti-PAK1 antibodies (diluted 1:500, Cell Signaling Technology, catalog number:2602T), anti-Collagen II alpha 1(Col2α1) antibodies (1:500, Abcam, catalog number: ab34712), anti-Aggrecan antibodies (1:500, Abcam, catalog number: ab36861), and anti-recombinant A disintegrin and metalloproteinase with thrombospondin 4 (ADAMTS4) antibodies (1:500, Abcam, catalog number: ab185722), anti-matrix metalloprotein 13 (MMP13) antibodies (1:500, Abcam, catalog number: ab219620), anti-interleukin-1β (IL-1β) antibodies (1:500, Abcam, catalog number: ab9722), anti-interleukin-6 (IL-6) antibodies (1:500, Abcam, catalog number: ab9324), anti-tumor necrosis factor-α (TNF-α) antibodies (1:500, Abcam, catalog number: ab6671) and anti-β-Actin antibodies (1:1000, Abcam, catalog number: ab8226), followed by incubation with horseradish peroxidase-conjugated secondary antibodies (1:5000, Abcam, catalog number: ab6728, ab6721). Electro-chemiluminescence plus reagent was used to expose the membrane in an enhanced chemiluminescence detection system (PerkinElmer, U.S.A.). All Western blots were repeated three times. The density of the bands on Western blots was quantified by densitometry analysis of the scanned blots using ImageJ software.

### RNA extraction and quantitative real-time PCR (qRT-PCR)

Total RNA was extracted using the TRIzol® reagent (Invitrogen, Paisley, U.K.). One microgram of total RNA was reverse transcribed using RevertAid First Strand cDNA Synthesis Kit (TaKaRa, Dalian, China), as detailed in the manufacturer’s guidelines. Quantitative PCR analysis was performed in a total volume of 20 μl containing template DNA, sense and antisenseprimers, SYBR® Green master mix (QIAGEN, Mississauga, Ontario, Canada). After incubation at 50°C for 2 min and at 95°C for 10 min, the mixtures were subjected to 40 amplification cycles. After PCR, a threshold cycle (*C*_T_ value) was obtained from each amplification curve. Relative mRNA expression was determined using the ΔΔC_T_ method, as detailed in the manufacturer’s guidelines (Applied Biosystems). The primers of PAK1, COL2α1, Aggrecan, ADAMTS4, MMP13, IL-1β, IL-6, TNF-α, and β-actin of rats were shown in Table 1.

**Table 1:** The primer sequences were used for qRT-PCR in the present study

Organism	Targets	Forward Primer/ Reverse Primer	Genbank accession No.
Human	*β-Actin*	Forward 5′-CATGTACGTTGCTATCCAGGC-3′	NM_001101
		Reverse 5′-CTCCTTAATGTCACGCACGAT-3′	
Human	*PAK1*	Forward 5′-CAGCCCCTCCGATGAGAAATA-3′	NM_002576
		Reverse 5′-CAAAACCGACATGAATTGTGTGT-3′	
Rat	*β-Actin*	Forward 5′-TGTCACCAACTGGGACGATA-3′	NM_031144
		Reverse 5′-GGGGTGTTGAAGGTCTCAAA-3′	
Rat	*PAK1*	Forward 5′- AGGCTGTTCTGGATGTTC-3′	NM_031010
		Reverse 5′- TATCGTCGTGTAGTCAGC-3′	
Rat	*Acan*	Forward 5′- GATCTCAGTGGGCAACCTTC -3′	NM_022190
		Reverse 5′- TCCACAAACGTAATGCCAGA -3′	
Rat	*Col2a1*	Forward 5′- CTCAAGTCGCTGAACAACCA -3′	NM_012929
		Reverse 5′- GTCTCCGCTCTTCCACTCTG -3′	
Rat	*ADAMTS4*	Forward 5′- TTCGCTGAGTAGATTCGTGG-3′	NM_023959
		Reverse 5′- CGGATTTGGGAGGCTTGC-3′	
Rat	*MMP-13*	Forward 5′-TGGCGACAAAGTAGATGCTG -3′	NM_133530
		Reverse 5′- TGGCATGACTCTCACAATGC -3′	
Rat	*IL-1β*	Forward 5′-TGTGATGTTCCCATTAGAC-3′	NM_031512
		Reverse 5′- ATTCGGTTGTTCACCATAA-3′	
Rat	*IL-6*	Forward 5′-CCTTCTTGGGACTGATGT-3′	NM_012589
		Reverse 5′-GTGGGTGTTGTCTGGTCA-3′	
Rat	*TNF-α*	Forward 5′-CCACGCTCTTCTGTCTACTG-3′	NM_012675
		Reverse 5′- CTCACTGTTCGGGCTCG-3′	

### Cell activity assay

Chondrocytes proliferative activity was assessed using a Cell Counting Kit-8 (CCK-8) (Dojindo). Briefly, equal numbers of chondrocytes (4 × 10^3^ cells/well) were seeded in a 96-well plate. Adherent chondrocytes were transfected with the siRNA-PAK1 and siRNA-NC under complete culture medium with 10 ng/ml IL-1β for 1, 3, 5 and 7 days. Ten microliters of CCK-8 solution was then added to each well of the plate according to the manufacturer’s instructions, and the plate was incubated at 37°C for 4 h in a humidified 5% CO_2_ atmosphere. The absorbance of each sample was measured at a wavelength of 450 nm.

### 5-ethynyl-29-deoxyuridine (EdU) incorporation assay

EdU incorporation assay was accomplished as previous reports [21]. Following designated treatment, chondrocytes were fixed with 2% paraformaldehyde for 15 min, followed by pulsing with EdU (Click-iT® Plus EdU Alexa-647® Imaging Kit, Life Technologies) for 2 h according to manufacturer’s protocol. Subsequently, cells were stained with 4′,6-Diamidino-2-phenylindole dihydrochloride (DAPI). Quantification of EdU^+^ chondrocytes (red cells) and DAPI^+^ chondrocytes (blue cell) was carried out using the cell counter plugin from the ImageJ software. The EdU incorporation rate was expressed as the ratio of EdU-positive cells to total DAPI-positive cells.

### Statistical analysis

Experimental results are expressed as mean ± SEM for at least three experiments. A two-sided Student’s *t*-test was used to calculate statistical significance. One-way analysis of variance (ANOVA) was performed to show the difference between groups. *P* < 0.05 was statistically significant.

## Results

### OA-related factors increase the expression of PAK1 in rat chondrocytes

**Figure 1:**
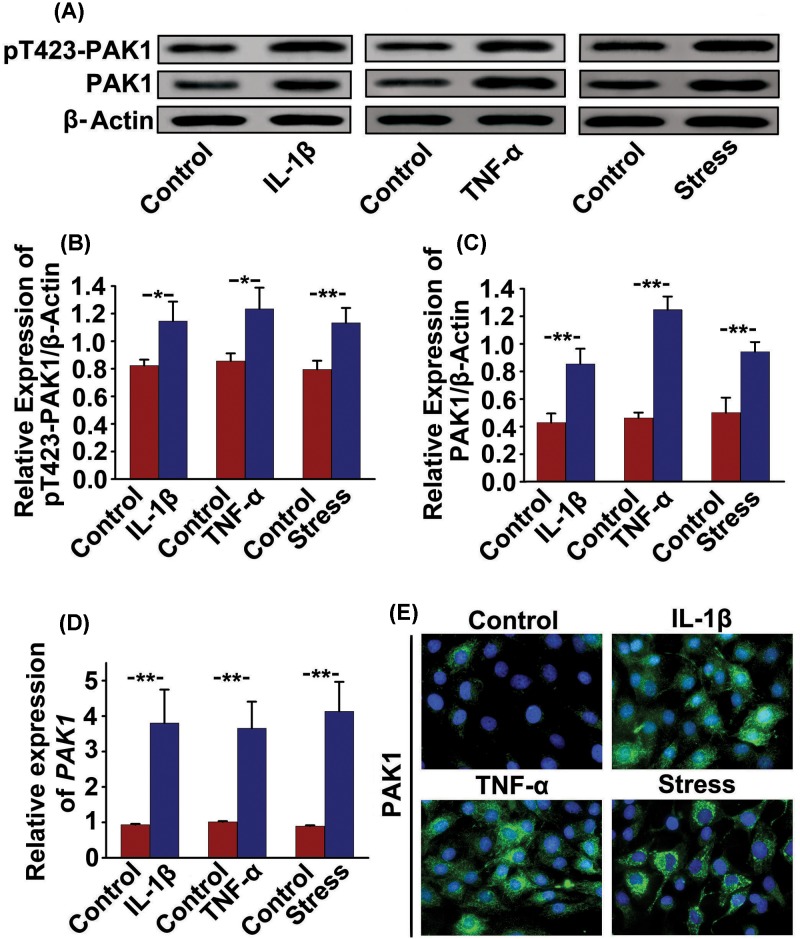
The OA-related factors increase the expression of PAK1 in rat chondrocytes (**A**) The protein expressions of pT423-PAK1 and PAK1 were examined by Western blot in rat chondrocytes treated with OA-related factors for 24 h. (**B** and **C**) Fold change relative to β-actin for the expression of these proteins were quantified by ImageJ software. *n* = 3, *P < 0.05, **P < 0.01 as compared with control. (**D**) The *mRNA* expressions of *PAK1* were measured by qRT-PCR in rat chondrocytes treated with OA-related factors for 24 h. *n* = 3, **P < 0.01 as compared with control. (**E**) Immunofluorescence staining showed that PAK1 was mainly located in cytoplasm and its fluorescence intensity was increased in rat chondrocytes treated with OA-related factors for 24 h; *n* = 5.

Whether the expressions of pT423-PAK1 and PAK1 are regulated by OA-related anabolic factors has not been defined. Therefore, we investigated the effects of OA-related factors, including 10 ng/ml IL-1β, 10 ng/ml TNF-α and 10% mechanical stress, on pT423-PAK1 and PAK1 expressions in rat chondrocytes. The results of Western blot showed that the protein expressions of pT423-PAK1 and PAK1 were significantly increased in rat chondrocytes treated with IL-1β (10 ng/ml) or TNF-α (10 ng/ml) or 10% mechanical stress (Figure 1A–C). Similarly, qRT-PCR analysis also confirmed that these OA-related factors obviously promoted *mRNA* expression of *PAK1* (Figure 1D). These results indicate that the expressions of pT423-PAK1 and PAK1 can be modulated by key factors affecting OA-related pathogenesis processes in rat cartilage. Next, immunofluorescence was used to observe the location and expression of PAK1 in rat chondrocytes exposed to OA-related factors. The results showed that PAK1 was mainly distributed in cytoplasm and its fluorescence intensity was enhanced by 10 ng/ml IL-1β, 10 ng/ml TNF-α and 10% mechanical stress respectively (Figure 1E).

### Aberrant activation of PAK1 in rats OA articular cartilage

**Figure 2:**
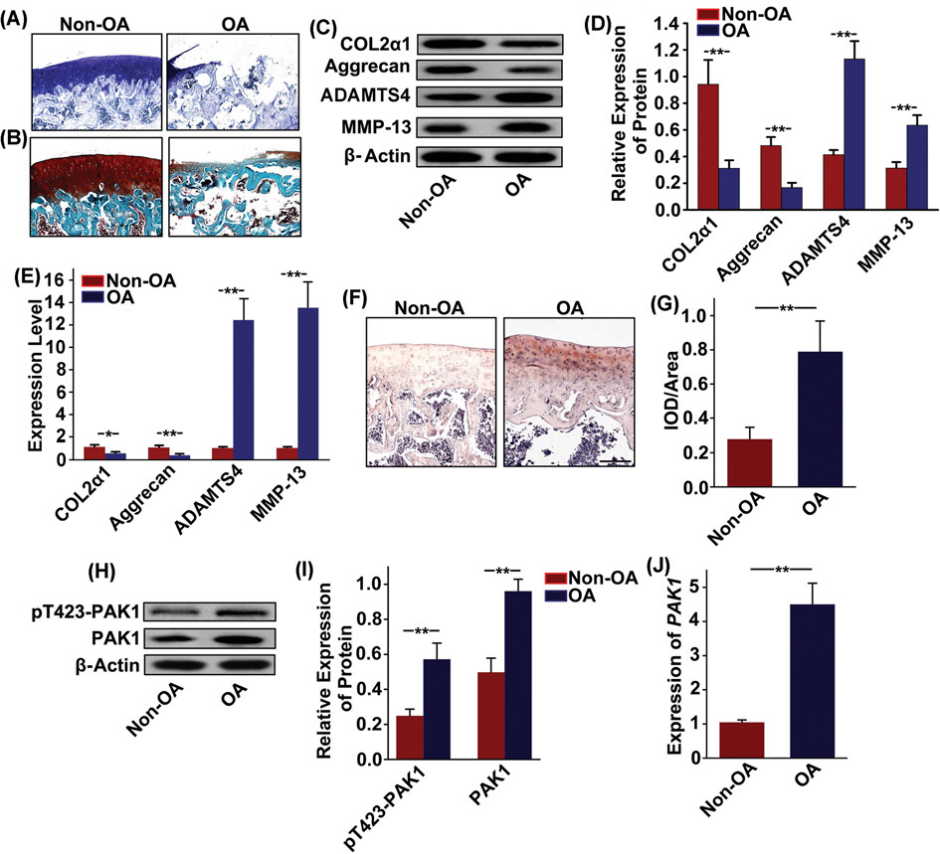
Aberrant activation of PAK1 in rats OA articular cartilage (**A** and **B**) Toluidine blue staining and Safranin-O/Fast Green staining in knee articular cartilage from OA rat showed that articular cartilage suffered serious destruction; *n* = 6. (**C**) The protein expressions of COL2α1, aggrecan, ADAMTS4, and MMP13 were measured by Western blot in articular cartilage obtained from Non-OA model rats and OA model rats. (**D**) Fold change relative to β-actin for the expression these proteins were quantified by ImageJ software; *n* = 3, **P < 0.01. (**E**) The *mRNA* expressions of *COL2α1, aggrecan, ADAMTS4* and *MMP13* were measured by qRT-PCR in articular cartilage obtained from Non-OA model rats and OA model rats; *n* = 3. *P < 0.05, **P < 0.01. (**F** and **G**) The expression of PAK1 was observed by immunohistochemistry staining in articular cartilage obtained from Non-OA model rats and OA model rats; scale bar = 200 µm; *n* = 4, **P < 0.01. (**H**) The protein expressions of pT423-PAK1 and PAK1 were measured by Western blot in articular cartilage obtained from Non-OA model rats and OA model rats. (**I**) Fold change relative to β-actin for the expression these proteins were quantified by ImageJ software; *n* = 3, **P < 0.01. (**J**) The *mRNA* expression of *PAK1* was measured by qRT-PCR in articular cartilage obtained from Non-OA model rats and OA model rats; *n* = 3, **P < 0.01.

To explore the relationship between PAK1 and OA pathogenesis, we compared the level of PAK1 in normal and OA rat articular cartilage. Toluidine blue staining and Safranin-O/Fast Green staining in knee articular cartilage from OA rat showed that articular cartilage suffered serious destruction (Figure 2A,B). Furthermore, decreased expressions of COL2α1 and aggrecan and increased expressions of ADAMTS4 and MMP13 were observed by Western blot (Figure 2C,D), and qRT-PCR analysis (Figure 2E) in articular cartilage from OA rats compared with specimens from Non-OA rats. Taken together, these results suggested that rat OA model was successfully established. Subsequently, patterns of PAK1 expression in rat articular cartilage were characterized by immunohistochemistry. The results showed that a large amount of brown PAK1 protein-positive expression was found in rat knee articular cartilage from OA group (Figure 2F,G). Western blot (Figure 2H,I) and qRT-PCR analysis (Figure 2J) also showed that the expression of PAK1 was significantly increased in rat knee articular cartilage from OA group.

### Increased expression of PAK1 in human OA articular cartilage

**Figure 3:**
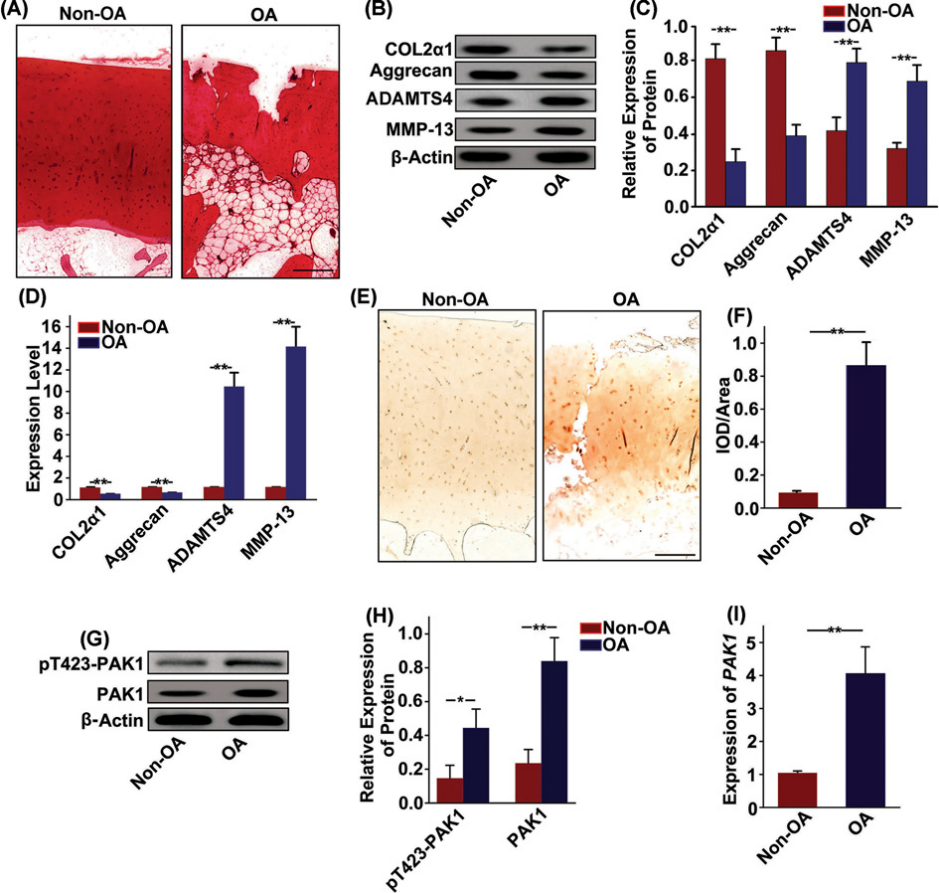
Increased expression of PAK1 in human OA articular cartilage (**A**) The articular cartilage samples obtained from patients with OA were stained with Safranin-O; scale bar = 200 µm, *n* = 3. (**B**) The protein expressions of COL2α1, aggrecan, ADAMTS4 and MMP13 were measured by Western blot in articular cartilage samples obtained from patients with OA. (**C**) Fold change relative to β-actin for the expression these proteins were quantified by ImageJ software; *n* = 3, **P < 0.01. (**D**) The *mRNA* expressions of COL2α1, *aggrecan, ADAMTS4* and *MMP13* were measured by qRT-PCR in articular cartilage samples obtained from patients with OA; *n* = 3, **P < 0.01. (**E** and **F**) The expression of PAK1 was observed by immunohistochemistry staining in articular cartilage samples obtained from patients with OA; scale bar = 200 µm, *n* = 4, **P < 0.01. (**G**) The protein expressions of pT423-PAK1 and PAK1 were examined by Western blot in articular cartilage samples obtained from patients with OA. (**H**) Fold change relative to β-actin for the expression these proteins were quantified by ImageJ software. *n* = 3, *P < 0.05, **P < 0.01. (**I**) The *mRNA* expression of *PAK1* was measured by qRT-PCR in articular cartilage samples obtained from patients with OA; *n* = 3, **P < 0.01.

The knee articular cartilage of patients with OA was used to further test the expression of PAK1. There was a serious destruction in the articular cartilage of patients with OA, as revealed by loss of Safranin-O staining (Figure 3A). Western blot (Figure 3B,C) and qRT-PCR analysis (Figure 3D) showed that the expressions of COL2α1 and aggrecan were obviously decreased, while the expressions of ADAMTS4 and MMP13 were significantly increased in articular cartilage from patients with OA. Next, patterns of PAK1 expression in OA patient’s articular cartilage were characterized by immunohistochemistry. The number of PAK1-positive cells was significantly increased in knee articular cartilage from patients with OA (Figure 3E,F). In addition, Western blot (Figure 3G,H) and qRT-PCR analysis (Figure 3I) also confirmed a significant up-regulation of PAK1 level in articular cartilage from patients with OA. Taken together, these results suggested that the expression of PAK1 was increased in articular cartilage from patients with OA.

### PAK1 alters the synthesis of molecules associated with cartilage degeneration in rat chondrocytes

**Figure 4:**
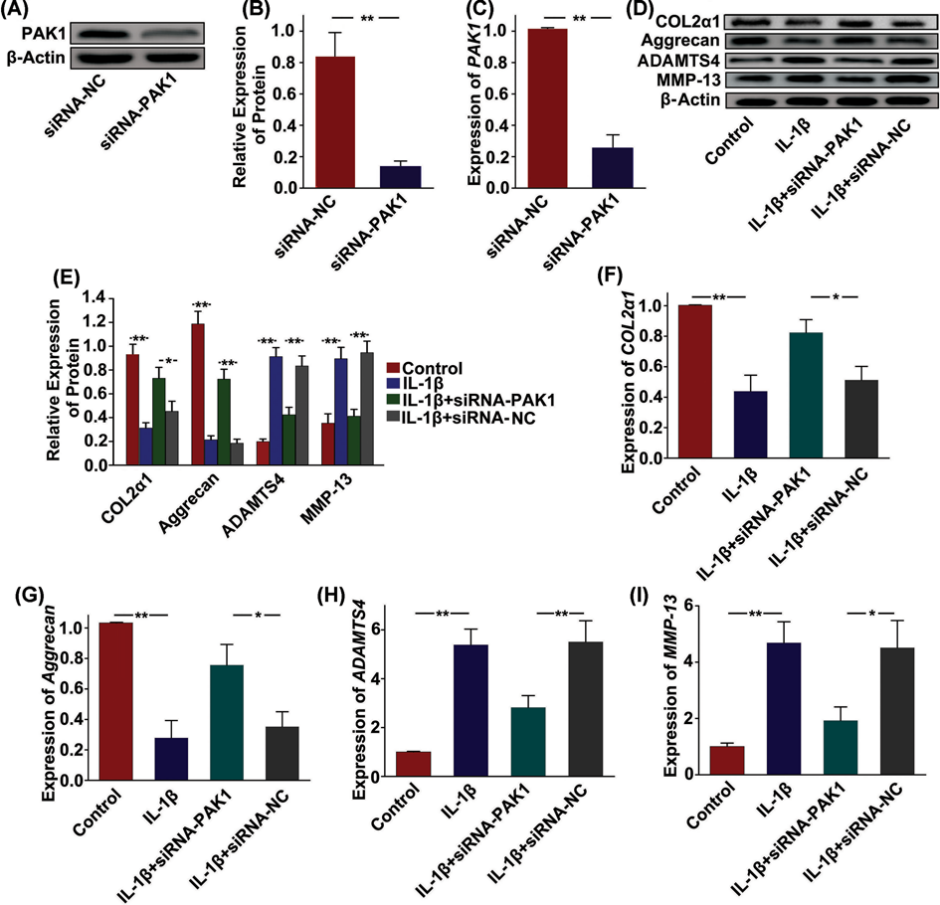
PAK1 alters the synthesis of molecules associated with cartilage degeneration in rat chondrocytes (**A**–**C**) The protein and mRNA expressions of PAK1 were measured by Western blot and qRT-PCR in rat chondrocytes transfected with siRNA-PAK1 for 24 h; *n* = 3, **P < 0.01. (**C**) The protein expressions of COL2α1, aggrecan, ADAMTS4 and MMP13 were measured by Western blot in chondrocytes transfected with siRNA-NC or siRNA-PAK1, followed by treating with 10 ng/ml IL-1β for 24 h; *n* = 3. (**E**) Fold changes relative to β-actin for the expression these proteins were quantified by ImageJ software; *n* = 3, *P < 0.05, **P < 0.01. (**F**–**I**) The *mRNA* expressions of *COL2α1, aggrecan, ADAMTS4* and *MMP13* were measured by qRT-PCR in rat chondrocytes transfected with siRNA-NC or siRNA-PAK1, followed by treating with 10 ng/ml IL-1β for 24 h; *n* = 3, *P < 0.05, **P < 0.01.

To address whether PAK1 modulated the production of ECM and cartilage degrading enzymes, PAK1 was silenced by siRNA-PAK1 in rat chondrocytes and then cellular ECM and cartilage degrading enzymes, including COL2α1, aggrecan, ADAMTS4 and MMP-13, were measured in *vitro.* The silencing effect of siRNA targeting PAK1 in rat chondrocytes was confirmed by Western blot (Figure 4A,B) and qRT-PCR analysis (Figure 4C). The results showed that the protein expressions of COL2α1 and aggrecan were obviously decreased in rat chondrocytes treated with 10 ng/ml IL-1β for 24 h. However, silencing of PAK1 could significantly alleviate the inhibitory effect of IL-1β on expressions of COL2α1and aggrecan (Figure 4D,E). Furthermore, IL-1β obviously increased the expressions of ADAMTS4 and MMP-13 in rat chondrocytes. PAK1 silencing attenuated IL-1β-induced up-regulation of ADAMTS4 and MMP-13 (Figure 4D,E). Similar results were also observed at the mRNA levels by qRT-PCR analysis (Figure 4F–I). Thus, these results suggested that PAK1 might modulate the synthesis of ECM and cartilage degrading enzymes in OA.

### PAK1 is involved in the regulation of human OA chondrocytes phenotype by IL-1β

**Figure 5:**
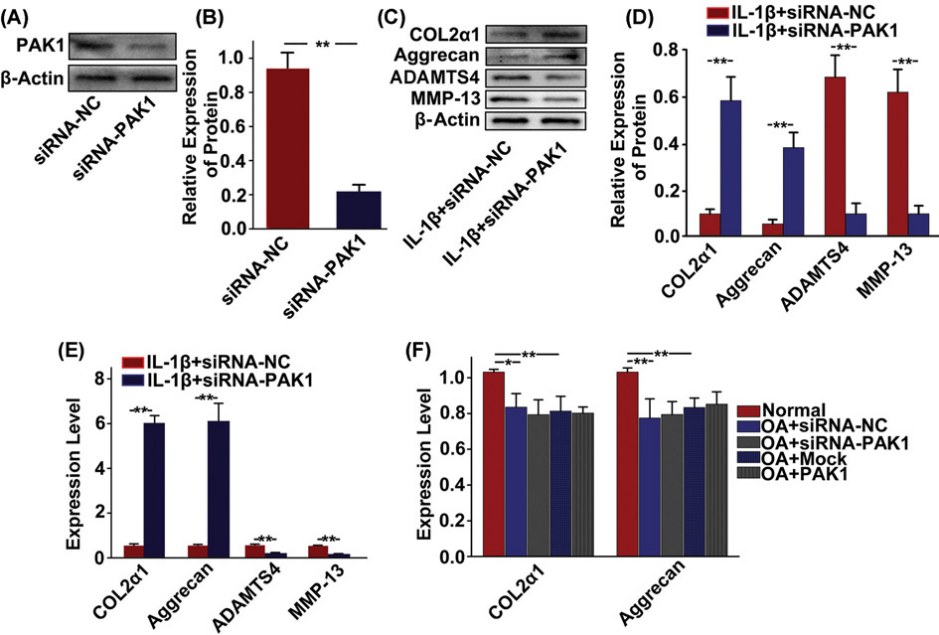
PAK1 is involved in the regulation of human OA chondrocytes phenotype by IL-1β (**A** and **B**) The protein expression of PAK1 was measured by Western blot in human OA chondrocytes transfected with siRNA-PAK1 for 24 h; *n* = 3, **P < 0.01. (**C** and**D**) The protein expressions of COL2α1, aggrecan, ADAMTS4 and MMP13 were measured by Western blot in human OA chondrocytes transfected with siRNA-NC or siRNA-PAK1, followed by treating with 10 ng/ml IL-1β for 24 h; *n* = 3, **P < 0.01. (**E**) The *mRNA* expression of *COL2α1, aggrecan, ADAMTS4* and *MMP13* were measured by qRT-PCR in human OA chondrocytes transfected with siRNA-NC or siRNA-PAK1, followed by treating with 10 ng/ml IL-1β for 24 h; *n* = 3, **P < 0.01. (**F**) The *mRNA* expression of *COL2α1* and *aggrecan* was measured by qRT-PCR in human OA chondrocytes transfected with siRNA or overexpression plasmid; *n* = 3, *P < 0.05, **P < 0.01.

To examine the potential role of PAK1 in the regulation of human OA chondrocytes phenotype by IL-1β, PAK1 was silenced by siRNA-PAK1 in human OA chondrocytes. The silencing effect of siRNA targeting PAK1 was confirmed by Western blot in human OA chondrocytes (Figure 5A,B). Silencing of PAK1 could significantly increase the expression of COL2α1 and aggrecan in IL-1β stimulated human OA chondrocytes. In addition, the expressions of ADAMTS4 and MMP-13 were down-regulated by siRNA-PAK1 in human OA chondrocytes stimulated with IL-1β (Figure 5C,D). Similar results were also confirmed by qRT-PCR (Figure 5E). To test whether PAK1 could affect the protein expressions of COL2α1 and aggrecan in human OA chondrocytes in absence of IL-1β stimulation, PAK1 was decreased with siRNA-PAK1 or increased with plasmid overexpressing PAK1. We found that silencing or overexpression of PAK1 in the absence of IL-1β did not significantly affect expressions of COL2α1 and aggrecan in human OA chondrocytes (Figure 5F), suggesting that PAK1 modulated the synthesis of molecules associated with cartilage degeneration in human OA chondrocytes under pathological conditions, but not under physiological conditions.

### PAK1 is involved in IL-1β-inhibited rat chondrocytes proliferation

**Figure 6:**
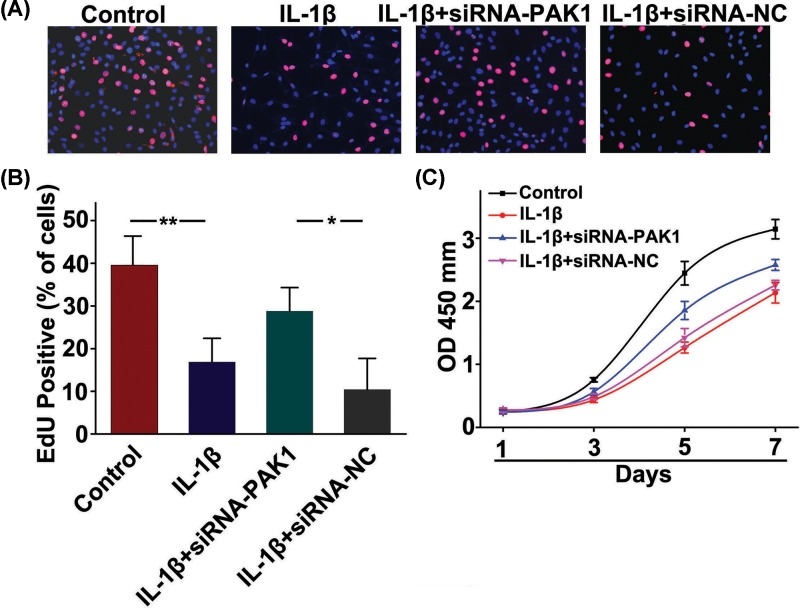
PAK1 is involved in IL-1β-inhibited rat chondrocytes proliferation (**A**) Representative photomicrographs of EdU staining. Red: EdU labeling of nuclei of proliferative cells (×200). (**B**) Quantitative data showing that the percentage of EdU-positive cells in different treatment groups (numbers of red vs numbers of blue nuclei); *n* = 6, *P < 0.05, **P < 0.01. (**C**) CCK-8 assay showed that cells viability was significantly inhibited in rat chondrocytes exposed to 10 ng/ml IL-1β from day 1 to day 7, whereas which was obviously alleviated by silencing of PAK1; *n* = 6.

To evaluate if PAK1 was involved in the inhibitory effect of IL-1β on rat chondrocytes proliferation, EdU staining was applied to observe the cells proliferation. We found that fewer cells with EdU-positive nuclei were showed in IL-1β stimulation group compared with the control group. However, silencing of PAK1 could increase the number of cells with EdU-positive nuclei in rat chondrocytes treated with 10 ng/ml IL-1β for 24 h, suggested that PAK1 was involved in IL-1β-inhibited chondrocytes proliferation (Figure 6A,B). Next, a CCK-8 assay was applied to monitor cell viability. The results showed that cells viability was significantly inhibited in rat chondrocytes exposed to 10 ng/ml IL-1β from day 1 to day 7, which was obviously alleviated by silencing of PAK1(Figure 6C).

### PAK1 activates inflammatory cytokines in rat chondrocytes stimulated with mechanical stress

**Figure 7:**
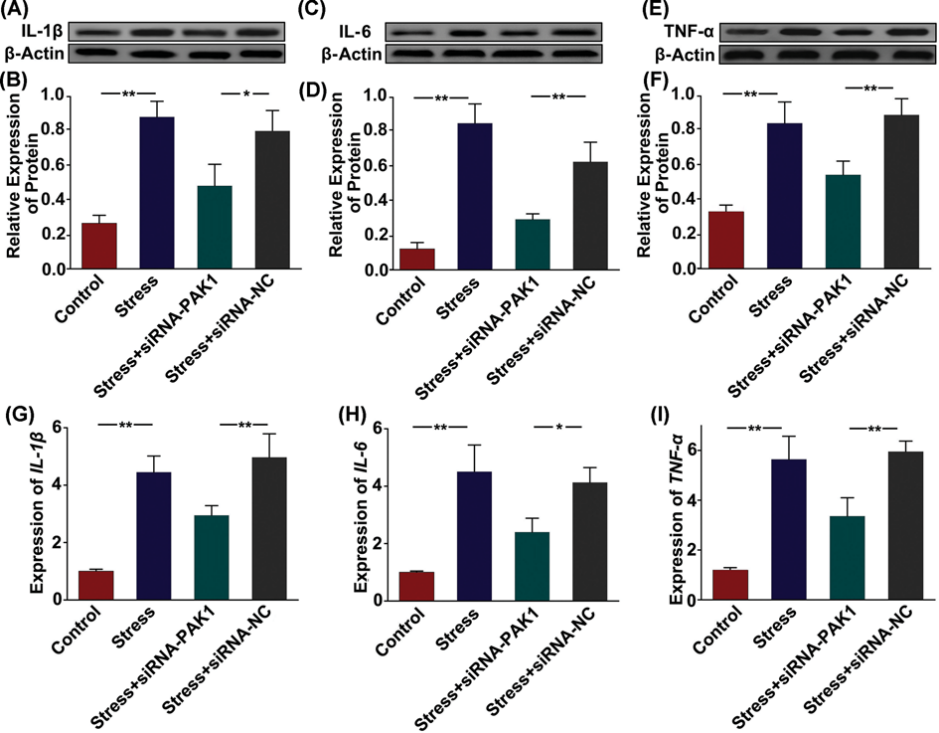
PAK1 activates inflammatory cytokines in rat chondrocytes stimulated with mechanical stress (**A**–**F**) The protein expressions of IL-1β (**A** and **B**), IL-6 (**C** and **D**) and TNF-α (**E** and **F**) were measured by Western blot in rat chondrocytes transfected with siRNA-NC or siRNA-PAK1, followed by treating with 10% mechanical stress for 24 h; *n* = 3, **P < 0.01, *P < 0.05. (**G**–**I**) The *mRNA* expressions of *IL-1β, IL-6* and *TNF-α* were measured by qRT-PCR in rat chondrocytes transfected with siRNA-NC or siRNA-PAK1, followed by treating with 10% mechanical stress for 24 h; *n* = 3, **P < 0.01, *P < 0.05.

To evaluate the role of PAK1 in inflammatory responses, we analyzed the alterations in the expression of inflammatory cytokines in rat chondrocytes stimulated with mechanical stress. The protein (Figure 7A–F) and mRNA levels (Figure 7G–I) of IL-1β, IL-6 and TNF-α were significantly increased in rat chondrocytes exposed to 10% mechanical stress. However, silencing of PAK1 could obviously alleviate the induction of IL-1β, IL-6 and TNF-α by mechanical stress in rat chondrocyte.

## Discussion

In the present study, we found that the expression of PAK1 was increased in rat chondrocytes treated with OA-related factors, which was also found to be significantly up-regulated in knee articular cartilage samples from patients with OA and OA model rats. The functional role of aberrant PAK1 expression was detected in the chondrocytes. We found that PAK1 overexpression inhibited the proliferation of chondrocytes. PAK1 activated inflammatory cytokines in chondrocytes stimulated with mechanical stress. Furthermore, our study confirmed that PAK1 modulated the production of ECM and cartilage degrading enzymes in chondrocytes. In the present study, we demonstrated that PAK1 might play an important role in the pathogenesis of OA. PAK1, a crucial regulator for chondrocyte function, might be used as therapeutic target for OA.

Previous studies have reported that PAK1 activation was responsible for initiation of inflammation [22]. Khare et al. found that overexpression of PAK1 promoted cell survival in inflammatory bowel diseases and colitis-associated cancer [23]. Dammann et al. confirmed that overexpression and activation of PAK1 in inflammation and colitis-associated cancer promoted nuclear factor kappa-B (NF-κB) activity via suppression of peroxisome proliferator-activated receptor γ in intestinal epithelial cells [24]. Zhou et al. found that TNF-α induced expression of MMP-9 through PAK1 in human skin keratinocytes, dermal fibroblasts and rat hepatic stellate cells [25]. In addition, Fu et al. found that activation of PAK1 was increased in rheumatoid arthritis fibroblast-like synoviocytes in response to TNF-a or IL-1β [26]. Even though PAK1 was involved in many inflammation-related diseases, very few studies reported that PAK1 was critical factor in the pathogenesis of OA.

In the present study, we confirmed that OA-related factors, including IL-1β, TNF-α and mechanical stress significantly increased the expression of PAK1 in chondrocytes. All these OA-related factors, IL-1β was recognized as the most important inflammatory cytokines in the pathogenesis of OA [15]. IL-1β was confirmed to induce inflammatory reactions and catabolic effect independently as well as being combined with other mediators with respect to the articular cartilage and other elements of joints [27]. Many studies showed that IL-1β inhibited the synthesis of the key structural proteins in chondrocytes, such as type-II collagen and aggrecan. In addition, IL-1β promoted the operation of chondrocytes in the synthesis of enzymes, including MMP-1, MMP-3, MMP-13 and ADAMTS-4, which have a destructive effect on cartilage components [28]. Our results also showed that IL-1β obviously decreased the expressions of aggrecan and COL2α1 in chondrocytes. Besides, IL-1β significantly induced the expressions of ADAMTS-4 and MMP-13, which were two critical enzymes for cartilage degrading in chondrocytes. These results were consistent with previous studies, suggesting that cells models used in the present study were reasonable and reliable.

PAK1 is widely distributed in the human body, which has been shown to interact with nicotinamide adenine dinucleotide phosphate (NADPH) oxidase in multiple cell types [10]. Previous studies showed that PAK1 was involved in the regulation of NADPH oxidase in diseases associated with inflammatory activation, proliferation and angiogenesis of endothelial cells [29,30]. In addition, PAK1 was also confirmed to control mitogen-activated protein kinase (MAPK), such as c-Jun N-terminal kinase (JNK) and p38 MAP kinases, and NF-κB activity [26]. Zhou et al. found that TNF-α or IL-1 treatment resulted in accumulation of PAK1 protein, which increased MMP-9 expression through JNK signaling pathway in human epithelial cells and fibroblasts [25]. In our study, IL-1β was confirmed to increase the expression of MMP13 through PAK1. However, the exact mechanisms by which PAK1 was involved in IL-1β-induced expression of MMP13 were unclear. It needs to be further explored in the future with further studies.

The proliferation of normal articular chondrocytes is important to maintain the number and function of articular chondrocytes. Chondrocyte proliferation has self-healing ability to repair joint damage [31]. An increasing amount of evidence has shown that proliferation and apoptosis of chondrocytes play great roles in cartilage development, aging and disease [32]. Our results showed that IL-1β markedly decreased proliferation of chondrocytes, which were consistent with previous studies [33]. Furthermore, we found that PAK1 was involved in IL-1β-inhibited proliferation of chondrocytes. Indeed, many studies have confirmed that PAK1 could regulate cells proliferation. Wang et al. found that overexpression of PAK1 promoted cell proliferation in cutaneous T-cell lymphoma via suppression of PUMA and p21 [34]. Fu et al. found that PAK1 promoted the proliferation and inhibited apoptosis of human spermatogonial stem cells via PDK1/KDR/ZNF367 and ERK1/2 and AKT pathways [30]. However, in the present study, we found that up-regulation of PAK1 by IL-1β inhibited proliferation of chondrocytes. It is possible that PAK1 might play different roles in different kinds of cells.

In the present study, we found that IL-1β significantly increased PAK1 expression in chondrocytes. However, overexpression of PAK1 also promoted the production of inflammatory cytokines in cartilage chondrocytes stimulated with mechanical stress. Therefore, positive feedback regulation between PAK1 and inflammatory factors was existed and may play an important role in the pathogenesis of OA. Our studies did not explore why PAK1 induced the production of inflammatory factors in chondrocyte. Previous studies showed that PAK1 could promote inflammatory pathways, such as signal transducers and activators of transcription 3 and NF-κB, which are associated with inflammation-associated diseases. [35] The exact mechanisms by which PAK1 induced the production of inflammatory factors in chondrocyte may be explored in the future studies.

In summary, our study provided new evidences that PAK1 might play an important role in the pathogenesis of OA. In the present study, we found that silencing of PAK1 could decrease the production of ECM and cartilage degrading enzymes. Furthermore, PAK1 was involved in IL-1β-inhibited proliferation of chondrocytes. Moreover, PAK1 activated inflammatory cytokines in cartilage chondrocytes stimulated with IL-1β. These findings suggested that PAK1 might be a novel target for control joint destruction of OA.

## Abbreviations

CCK-8: Cell Counting Kit-8
DAPI: 4′,6-Diamidino-2-phenylindole dihydrochloride
ECM: extracellular matrix
EdU: 5-ethynyl-29-deoxyuridine
GTP: guanosine triohosphte
JNK: c-Jun N-terminal kinase
MAPK: mitogen-activated protein kinase
NADPH: nicotinamide adenine dinucleotide phosphate
OA: osteoarthritis
PAK1: P21-activated kinase-1
